# Correlation Between Body Mass Index and Dental Caries Among Three- to 12-Year-Old Schoolchildren in India: A Cross-Sectional Study

**DOI:** 10.7759/cureus.5421

**Published:** 2019-08-18

**Authors:** Kavitha Swaminathan, Vasanthakumari Anandan, SelvaKumar H, Eapen Thomas

**Affiliations:** 1 Pedodontics and Preventive Dentistry, Sri Ramachandra Institute of Higher Education and Research, Chennai, IND; 2 Paediatric Dentistry, Adhiparasakthi Dental College and Hospital, Melmaruvathut, IND; 3 Pediatric Dentistry, Dr. Sunny Medical Centre, Sharajah, ARE

**Keywords:** obesity, school children, over weight, dental caries, body mass index

## Abstract

Introduction: Diet is a deeply ingrained element of a person’s life. Children’s dietary habits are a significant contributor to obesity and dental caries. Dental caries during childhood continues to be a significant public health concern, while childhood obesity is increasingly being cited as a major public health problem. This study aimed to assess the correlation between body mass index (BMI) and dental caries in children aged three to 12 years who attended both government and private schools in Chennai, Tamil Nadu, India.

Materials and methods: We conducted a cross-sectional review of 2200 children aged three to 12 years with clinically recorded dental caries. The World Health Organization diagnostic criteria for BMI percentile was used to evaluate and record dental caries clinically. The Mann-Whitney and the Kruskal-Wallis tests were used for univariate comparisons.

Results: Mean values between the overweight category and underweight category revealed no significant differences.

Conclusion: We found no association between BMI-for-age and dental caries in children in both primary and mixed dentition. This relationship should be investigated further by longitudinal studies.

## Introduction

Obesity and dental caries are multifactorial conditions, both having comprehensive etiology and factors such as dietary habits and available nutrients, oral hygiene, or saliva [1]. Studies have shown that excessive food intake or inadequate physical activity are underlying causes of obesity [2]. The interaction of genetic, environmental, and behavioral factors could lead to childhood obesity has been reported [3]. Changes in diet and lifestyle, such as an increase in wealth and access to carbohydrate-rich, high-calorie food and drinks could be attributed to the increase in the prevalence of both dental caries and obesity [4]. The prevalence of obesity has increased in a magnitude of two to five times in developed countries, and almost four times in developing countries [5]. Several studies to date have evaluated the relationship between tooth decay and obesity; however, results are sometimes contradictory [6-9]. Although some studies have reported that the prevalence of obesity in India is high, its relationship with dental caries appears to be scant [10, 11]. Hence, this study assessed the correlation between age-specific body mass index (BMI) and dental caries in children aged three to 12 years.

## Materials and methods

A cross-sectional observational study was conducted among school-going children aged three to 12 years in Chennai, India. Both government and private schools were chosen for the study so that children from all socioeconomic levels would be covered. Ethical approval and safeguarding of all principles of the Helsinki declaration were obtained from the Sri Ramachandra Institute of Higher Education and Research in Chennai. Written informed consent for involvement in the study was obtained from each child's parent or guardian.

One government and one private school were randomly selected, and a pilot study was conducted. The prevalence of dental caries was 69% as observed in our pilot study, based on which the sample size was determined (1067) using the Epi Info™ Version 6 (Centers for Disease Control and Prevention, Atlanta, US) statistical package at 95% confidence interval. Eight schools (four government and four private schools) were included in the study, and all children from each class were examined. Thus, 2200 children aged three to 12 years were examined. The children were then divided into two groups based on their age: Group I Primary Dentition (ages three to five years) and Group II Mixed Dentition (ages six to 12 years).

Children with long-standing systemic illness, physical or mental disability or those under any medication within the past two months were excluded from the study. A trained examiner - a person qualified for clinical assessment during numerous educational and clinical sessions in the Department of Pedodontics and Preventive Dentistry - examined the children for the presence of dental caries. All instruments were sterilized and packed (one pack per sample) in sufficient quantities for each day of work.

Children were instructed to stand straight, and their height and weight were recorded while wearing light clothing and no shoes. Height was measured using a standard height chart and weight using an analog weighing scale. All findings were entered in a data sheet for further analysis.

BMI was calculated using the height and weight recorded, and the formula used to calculate BMI was weight in kg/(height in m2).

Children in each age group were further divided into four subgroups based on their BMI-for-age-as developed by the National Centre for Health Statistics in collaboration with the National Centre for Chronic Disease Prevention and Health Promotion: 1) underweight, meaning a BMI-for-age less than the fifth percentile; 2) normal, meaning a BMI-for-age greater than or equal to the fifth percentile and less than the 85th percentile; 3) at risk of overweight, meaning a BMI-for-age greater than the 85th percentile but less than the 95th percentile; and 4) overweight, meaning a BMI-for-age greater than the 95th percentile [12]. None of the children in this study belonged to the “at risk of overweight” category; therefore, this group was omitted.

The evaluation of dental caries was followed by diagnostic criteria suggested by the World Health Organization (WHO) [13]. A modified WHO proforma was used to record data regarding the general information and Dunning type III clinical examination was used for examination [14]. An intraoral examination was done visually with the aid of a mouth mirror and probe, and the findings were recorded as decayed, missing, or filled using the (decayed-missing-filled teeth [DMFT]/decayed- extracted-filled teeth [DEFT]) index. Since children were in different stages of the mixed dentition, naturally exfoliated primary teeth were not taken into consideration.

Kappa statistic was used to determine the intra-examiner reproducibility, which was 0.85. The intra-examiner reproducibility was assessed by asking the teacher to randomly select five students who were examined the preceding day, and these children were reinspected.

The data collected were tabulated and analyzed using SPSS for Windows, Version 13.0. (SPSS Inc., Chicago, US). However, one-way analysis of variance and Kruskal-Wallis tests were used for correlating obesity and dental caries; p < 0.05 was considered statistically significant, and p < 0.001 was considered highly statistically significant.

## Results

Of the total study population, 33.2% of the children were three to five years old (Group I), and 66.8% were six to 12 years old (Group II). Of those in the three to the five-year-old group, 52.6% were boys, and 47.3% were girls, while 51.9% were boys and 48.1% were girls in the six to 12-year-old group. In Group I, 45.1% of the children attended government school, and 54.9% attended private school, as compared to Group II, where 45.0% of the children attended government school, and 55.0% attended private school (Table 1).

**Table 1:** Distribution of subjects according to gender and schools

Group	Schools	Male (n)	%	Female (n)	%	Total (n)	%
Group I (3-5 years)	Government	179	46.6%	150	43.4%	329	45.1%
	Private	205	53.4%	196	56.6%	401	54.9%
	Total	384	100.0%	346	100.0%	730	100.0%
Group II (6-12 years)	Government	351	46.0%	318	45.0%	669	45.0%
	Private	412	54.0%	389	55.0%	801	55.0%
	Total	763	100.0%	707	100.0%	1470	100.0%

Table 2 shows that among the 730 children in the three to five-year-old group, 594 (81.4%) were in the normal weight category, 78 (10.7%) were overweight, and 58 (7.9%) were underweight. Among the 1470 children in the six to 12-year-old group, 958 (65.2%) had a normal weight, 220 (14.9%) were overweight, and 229 (19.9%) were underweight.

**Table 2:** Distribution of subjects according to BMI and school category BMI - body mass index

Group	BMI	Government (n)	%	Private (n)	%	Total (n)	%
Group I (3-5 years)	Overweight	28	8.4%	50	12.5%	78	10.7%
	Normal weight	279	84.3%	315	79.0%	594	81.4%
	Underweight	24	7.3%	34	8.5%	58	7.9%
	Total	331	100.0%	399	100.0%	730	100.0%
Group II (6-12 years)	Overweight	78	11.7%	142	17.7%	220	14.9%
	Normal weight	484	72.3%	474	59.2%	958	65.2%
	Underweight	107	16%	185	23.1%	292	19.9%
	Total	669	100.0%	801	100%	1470	100.0%

The mean DMFT/DEFT value in Group I (4.26 ± 3.81) was significantly higher than the mean value in Group II (2.47 ± 2.20; p < 0.001). A Mann-Whitney U test was used to calculate the p-value (Table 3).

**Table 3:** Mean, standard deviation and test of significance of mean values between Group I (age 3-5 years) and Group II (age 6-12 years) SD - standard deviation

Group	Mean ± SD	p-value
Group I (3-5 years)	4.26 ± 3.81	< 0.001
Group II (6-12 years)	2.47 ± 2.20	< 0.001

Table 4 shows BMI and dental caries in both age groups. The mean value in the overweight group was significantly lower than the normal weight group (p < 0.001). Further, the mean value in the underweight group (2.74 ± 3.92) was significantly lower than the normal weight group (p < 0.001). However, there was no significant difference in mean values between the overweight category and the underweight category (p = 1.00). The Kruskal-Wallis one-way analysis of variance was used to calculate the p-value.

**Table 4:** BMI and dental caries BMI - body mass index DMFT - decayed-missing-filled teeth SD - standard deviation

Group	BMI	Mean DMFT	SD	p-value
Group I (3-5 years)	Overweight	1.85	2.08	< 0.001
	Normal weight	4.73	3.81	
	Underweight	2.74	3.92	
Group II (6-12 years)	Overweight	1.00	0.00	< 0.001
	Normal weight	2.42	2.09	
	Underweight	4.41	3.86	

## Discussion

Childhood obesity is a public health problem with increasing importance in the urbanized world [15]. Being overweight/obese comes with numerous health problems [16]. Studies exploring the relationship between caries experience and BMI reported conflicting results [6-7, 17-18]. The objective of this study was to investigate the relationship between BMI and dental caries prevalence among schoolchildren aged three to 12 years.

In the present study, in both Group I (81.4%) and Group II (65.2%), the majority of children were in the normal weight group. In a recent obesity update, the percentage of the population with obesity by age 15 in India is 5% whereas in the United States of America it is 38.2% [19].

Dietary deficiency occurring early in the life of a child, when the primary teeth are being formed, will enhance the occurrence of caries three to four years later [20]. The ability of a tooth to withstand caries attack is reduced if the teeth experienced nutritional harm during the essential stages of their growth [21]. Acs et al. reported that children with early childhood caries weighed significantly less than age- and sex-matched caries-free children [22]. In the present study, in Group I, underweight children showed a mean DEFT value of 2.74 ± 3.92, which was significantly lower than the normal weight group (4.73 ± 3.81). In Group II, underweight children had a mean value of 4.41 ± 3.86, whereas normal-weight children had a lesser DMFT value of 2.42 ± 2.09. This difference was statistically significant in both groups.

In our study, there is no difference in caries experience between normal weight and overweight children. This finding is consistent with studies done by Tuomi [23], Chen et al. [24], Moreira et al. [7], and Assi et al. [25].

With the known culture difference, an Indian diet is different from a western diet. Contradicting the results of our study - in which primary teeth had less caries experience in underweight children than normal-weight children - a study in Peruvian children reported that those who were malnourished had higher dental caries prevalence in primary dentition and delay in tooth eruption [26]. There was a positive association between obesity and dental caries in the permanent dentition but the causative direction of this relationship is unclear reported in a systemic review [27].

Our study had several limitations. Because overweight children are not very common in India, as compared to Western countries, it is difficult to collect a sufficient number of children who are overweight in a particular sample population. Moreover, the BMI values that were used were not put forth based on the Indian population. This could have resulted in a variation in the distribution of samples, as many of the normal-weight children would fall within the overweight category if the BMI values were given specific of the Indian population. Another limitation of the study, which may have underestimated caries experience, is that bitewing radiographs were not used to identify proximal caries. Initial proximal caries detected by bitewing radiographs would have altered the mean caries experience in all groups.

## Conclusions

Overall caries experience is higher in primary dentition compared to the mixed dentition. Overweight children had less caries experience than the other two subgroups in both primary and mixed dentition. In the primary dentition, both underweight and overweight children had less caries experience than normal weight children. In mixed dentition, underweight children showed a greater caries experience than overweight and normal weight children. The results from this study demonstrated that no association could be assigned between BMI-for-age and dental caries; therefore, advance research is essential to addressing factors specific to overweight in children and preventing dental caries in primary and mixed dentition. A study involving a larger sample with radiographic investigations is needed in the future to further evaluate the correlation between BMI and dental caries.
